# Working Mechanisms of Triple-Oxide Mesoporous Hole-Transport-Layer-Free Printable Perovskite Solar Cells via Impedance Spectroscopy

**DOI:** 10.1021/acs.jpclett.5c01405

**Published:** 2025-08-08

**Authors:** Pablo F. Betancur, Maayan Sohmer, Iván Mora-Seró, Lioz Etgar, Pablo P. Boix

**Affiliations:** † 253325 Instituto de Ciencia de los Materiales de la Universidad de Valencia (ICMUV), 46980 Paterna, València Spain; ‡ Institute of Chemistry, Casali Center for Applied Chemistry, The Center for Nanoscience and Nanotechnology, 26742 The Hebrew University of Jerusalem, Jerusalem 91904, Israel; § Institute of Advanced Materials (INAM), 16748 Universitat Jaume I (UJI), Avenida de Vicent Sos Baynat, s/n, 12071 Castelló, Spain; ∥ Instituto de Tecnología Química, 16379 Universitat Politècnica València−Consejo Superior de Investigaciones Científicas, Avinguda dels Tarongers, 46022 València, Spain

## Abstract

All-printed mesoporous perovskite solar cells (PSCs) show great potential for scalable photovoltaic technologies, yet direct identification of their key working mechanisms by impedance spectroscopy (IS) is not well-established. IS response of printable hole transport layer (HTL)-free triple mesoporous (mp) TiO_2_/ZrO_2_/ITO PSCs with varying TiO_2_ electron transport layer (ETL) thicknesses (500–1200 nm) reveals strong interplay between the mesoporous scaffold architecture and charge carrier dynamics, significantly impacting resistive and capacitive features of the devices. The emergence of an intermediate-frequency feature can be related to chemical capacitance of the mp-TiO_2_ layer, a phenomenon commonly associated with dye-sensitized solar cells, decoupling recombination, and key transport phenomena for both charge carriers. An updated equivalent circuit model, incorporating chemical capacitance and associated transport/recombination resistances can capture these effects. These findings provide valuable insights into the role of mesoporous scaffold engineering in printable PSCs and offer a robust characterization tool for optimizing scalable photovoltaic architectures.

## Graphical abstract

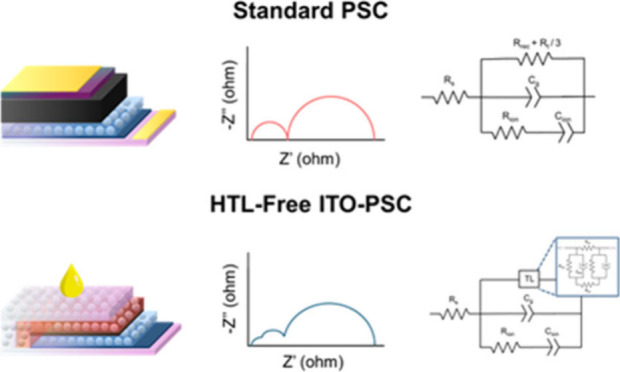

Halide perovskites are a family of emerging photovoltaic materials that have gained major attention in recent years due to their low-cost fabrication, versatility in device architecture, and rapid rise in efficiency. For commercial implementation, scalable manufacturing strategies for perovskite solar cells (PSCs) are essential. Several large-area fabrication techniques, including inkjet printing,^200^ screen printing,^1^ and roll-to-roll printing,^2^ have been explored for PSC production, offering compatibility with existing industrial processes. Among fully printable approaches, carbon-based perovskite solar cells (C-PSCs) form a particularly interesting approach that avoids noble metal evaporation and, in some cases, enables the fabrication of hole transport layer (HTL)-free devices.^3–6^ A clear example of such C-PSC architecture is the triple mesoporous structure (TiO_2_, ZrO_2_, and carbon).^7,8^ In this system, TiO_2_ acts as an electron transport layer (ETL), while no specific material acting as a HTL is present. The perovskite infiltrates the mesoporous scaffold and crystallizes within it, forming highly stable devices thanks to the hydrophobic properties of carbon, which serves as a contact. At the same time, the ZrO_2_ layer prevents shortcuts between the ETL and carbon. Recently, a unique concept was developed involving the use of a mesoporous indium tin oxide (ITO) layer to replace the carbon electrode,^9^ opening the way to operate in a bifacial configuration (ITO-PSCs).^10–12^ This structure offers unique advantages, including recyclability, as the inorganic mesoporous stack (TiO_2_, ZrO_2_, and ITO) remains intact even after perovskite degradation. The ITO transparency enables light absorption from both the front and back electrodes, maximizing power output and improving the device versatility. However, HTL-free architectures often suffer from increased carrier recombination at the perovskite/electrode interface, with the ETL being a critical element in determining overall device performance.^5,6^ Therefore, understanding the impact of the conduction band edge, defects, and morphology of the ETL on the charge carrier dynamics of the device is crucial to achieve efficiency comparable to the planar architecture.

In this context, impedance spectroscopy (IS) is a non-destructive electrical characterization technique widely used for analyzing PSCs under operating conditions. This method in the frequency domain applies a small alternating current (AC) signal perturbation around a fixed direct current (DC) voltage bias, enabling the extraction of charge carrier dynamics occurring in different time (frequency) regimes. By analysis of these processes individually, IS provides valuable insights into key processes, such as charge transport, recombination losses, interfacial charge accumulation, and ionic migration, among other physical phenomena occurring in the devices. However, the IS spectra interpretation is challenging, as multiple overlapping processes contribute to the measured response. Particularly, in the context of PSCs, ionic contributions are also present alongside the electron response. To extract meaningful physical parameters, IS data are typically fitted using equivalent circuit models, which translate complex impedance spectra into electrical components related to specific physical phenomena. While the equivalent circuit analysis of PSC has presented significant advances, there is still ongoing discussion on how to approach IS spectra beyond the characteristic two-arc shape typically observed in these devices.

Here, we analyze the IS response of a HTL-free ITO-PSC as a case study to investigate the conditions under which the Fermi level of the ETL plays a key role in the device response. We build upon established equivalent circuit models used to interpret the IS response of conventional planar PSCs, extending them to account for the additional capacitive and resistive effects observed in the ITO-PSC architecture. Interestingly, under certain circumstances, this kind of PSC adopts a characteristic working mechanism of dye-sensitized solar cells. This approach allows identification of the dominating role of the quasi-Fermi levels, which determine the main working mechanisms of the device and the performance-limiting factors.

The samples analyzed were prepared on transparent conducting glass substrates coated with fluorine-doped tin oxide (FTO). The ITO-PSC configuration features a printed triple mesoporous (mp) structure, consisting of sequentially screen-printed layers of mp-TiO_2_, mp-ZrO_2_, and mp-ITO. Perovskite precursor solutions deposited onto the mp-ITO layer percolate through the different mesoporous layers and crystallize within the pores, following a previously established protocol.^10^ The device structure is illustrated schematically in Figure 1a, with a cross-sectional focused ion beam (FIB) image provided in Figure 1b. The perovskite employed in this work is a mixed-cation, mixed-halide composition: FA_0.85_MA_0.15_Pb­(I_0.85_Br_0.15_)_3_ (where FA represents formamidinium and MA represents methylammonium). For comparison, a reference n–i–p cell with an ITO/mp-TiO_2_/perovskite/spiro-MeOTAD/Au configuration is also included, as shown in Figure 1d; see Figure 1e for a cross-sectional SEM image and Figure S1 for current density–voltage (*j*–*V*) characteristics. In this configuration, spiro-MeOTAD acts as a HTL and Au acts as a hole extracting layer.

**Figure 1:**
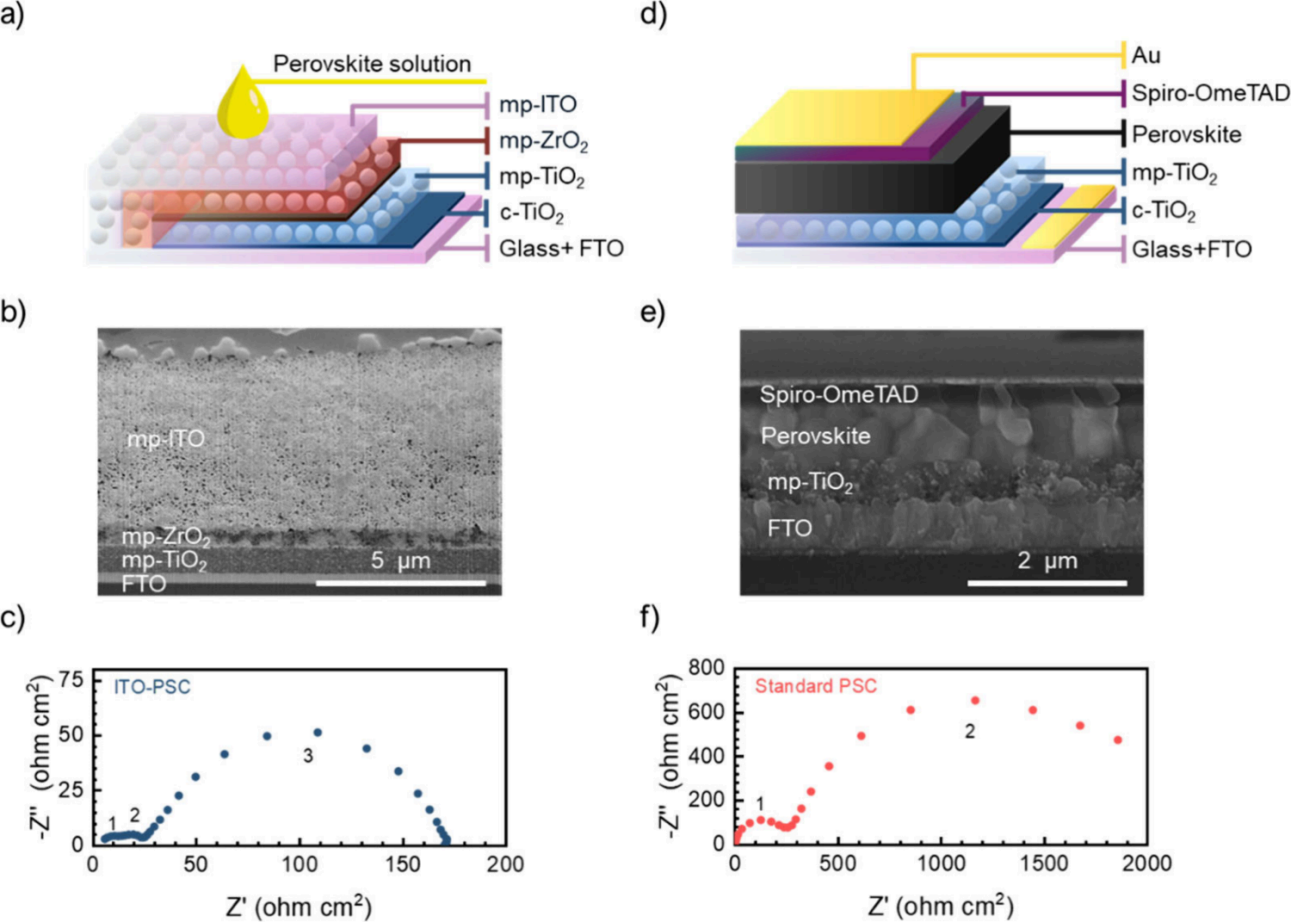
Schematic of (a) ITO-PSC with a triple-oxide mesoporous structure and (d) standard PSC with a mp-TiO_2_ layer. (b) SEM cross-section view images of the ITO-PSC cell with a mp-TiO_2_ thickness of 800 nm and (e) standard PSC with a mp-TiO_2_ thickness of 300 nm. (c) Nyquist plots of ITO-PSC and (f) standard PSC configuration at 0.1 sun illumination and 0.5 V, with the different observed arcs in both configurations numbered.

The standard cell architecture with mp-TiO_2_ combined with various perovskite compositions has been extensively studied using IS.^13^ The Nyquist plot of the PSC impedance spectrum typically exhibits one or two distinct semicircles, as displayed in the representative plots in Figure 1. For the two-arc spectra, the high-frequency (HF) response, occurring in the kHz range, is dominated by geometrical capacitance and electronic processes. In contrast, the low-frequency (LF) physical origin is related to ionic diffusion processes, typically occurring in the Hz to mHz range.

However, with the incorporation of the triple-oxide mesoporous structure, more complex dynamics emerge within the cell, resulting in a three-arc IS spectral response; compare Figure 1c and f for reference spectra at 0.1 sun illumination and 0.5 V applied voltage. This Nyquist shape has been observed previously in PSCs and is often attributed to distinct processes occurring at different frequency ranges. For instance, the LF arc is often attributed to ion modulation within perovskite grains, influenced by the material’s composition and local distortions, while the mid-frequency (MF) response has been associated with ion diffusion along grain boundaries and interfaces.^14–16^ An alternative approach suggests that the three arches correspond to processes taking place at specific layers and interfaces of the device, attributing the HF to charge exchange at the hole-selective layer/metal interface. The MF feature reflects charge carrier recombination, such as interfacial recombination of electrons in TiO_2_ with holes in the perovskite. Meanwhile, the LF response is associated with ionic movement within the perovskite material.^17^

Here, the reference cell, based on a standard architecture, exhibits two distinct features in the Nyquist plot of the IS spectrum; see Figure 1f. However, for the ITO-PSC configuration, a MF feature emerges; see Figure 1c, suggesting a direct link between the device architecture and the observed spectral response. Given that both configurations share the same perovskite composition, the evolution of spectral features is more likely governed by the modification in the device structure rather than intrinsically absorbed material properties. While the architecture includes a mp-ZrO_2_ layer, its influence on the key phenomena observed in the IS spectra of the devices is considered negligible. The primary role of mp-ZrO_2_ in these architectures is to act as a wide-bandgap, electrically inactive scaffold that prevents charge recombination between mp-TiO_2_ and mp-ITO. Consequently, while this dielectric material has its own impedance signature, any significant capacitive influence is expected only at very high frequencies (>1 MHz), well outside the range of interest for this work. Furthermore, since the mp-ZrO_2_ layer is parallel to the perovskite, the dominant electrical response in the analyzed frequency range is expected to come from the perovskite layer itself. Therefore, we focus our analysis on how the electron transport layer thickness affects the impedance response.

A systematic variation of the mp-TiO_2_ thickness from 500 to 1200 nm can be used to further explore its impact on the impedance response. Figure 2a presents the current–voltage (*j*–*V*) curves of the devices, indicating that the highest performance is achieved with a mp-TiO_2_ thickness of 800 nm. The observed improvement in device performance from 500 to 800 nm of mp-TiO_2_ aligns with simulation studies reported in the literature.^5,6^ This performance enhancement from 500 to 800 nm devices is attributed to the formation of a thicker absorbing layer with reduced shunting between electron and hole contacts. However, further increases in the thickness restrict perovskite infiltration within the mesoporous structure, leading to higher charge recombination across the mp-TiO_2_ and perovskite interface, negatively impacting photoconversion efficiency (PCE).^18,19^

**Figure 2:**
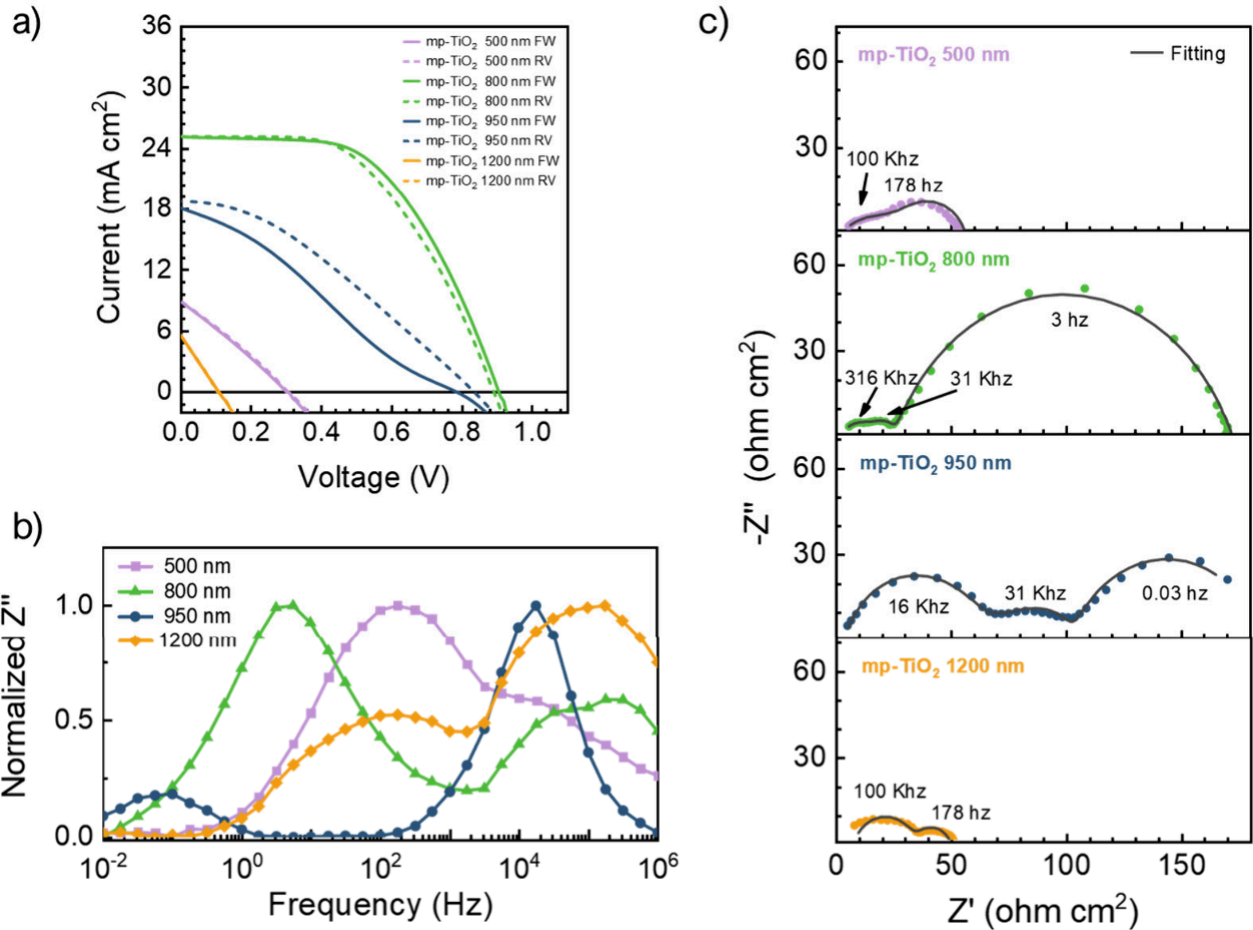
(a) *j*–*V* curves of ITO-PSCs with different mp-TiO_2_ thicknesses at 1 sun illumination. (b) Normalized Bode plot of the devices at the open circuit condition. (c) Nyquist plots of the devices, presented at voltages where the features of the impedance spectra are clearly distinguishable: 800 and 950 nm devices at 0.5 V and 500 and 1200 nm devices at *V* = *V*_oc_. Frequency values corresponding to key features are highlighted. The IS measurements were taken at 0.1 sun illumination. The fittings were performed using the equivalent circuit described in Figure 3f, with the following parameters. (i) mp-TiO_2_ 500 nm: *R*_s_ ≈ 30 Ω, *R*_rec_ ≈ 540 Ω, *R*_tr_ ≈ 290 Ω, *C*_MF_ ≈ 10^–6^ F, *C*_g_ ≈ 10^–10^ F, *R*_ion_ ≈ 390 Ω, and *C*_ion_ ≈ 8 × 10^–6^. (ii) mp-TiO_2_ 800 nm: *R*_s_ ≈ 30 Ω, *R*_rec_ ≈ 500 Ω, *R*_tr_ ≈ 1280 Ω, *C*_MF_ ≈ 6 × 10^–7^ F, *C*_g_ ≈ 10^–9^ F, *R*_ion_ ≈ 225 Ω, and *C*_ion_ ≈ 5 × 10^–5^. (iii) mp-TiO_2_ 950 nm: *R*_s_ ≈ 30 Ω, *R*_rec_ ≈ 2801 Ω, *R*_tr1_ ≈ 890 Ω, *R*_tr2_ ≈ 440 Ω, *C*_MF_ ≈ 5 × 10^–5^ F, *C*_g_ ≈ 10^–7^ F, *R*_ion_ ≈ 1850 Ω, and *C*_ion_ ≈ 8 × 10^–4^. (iv) mp-TiO_2_ 1200 nm: *R*_s_ ≈ 70 Ω, *R*_rec_ ≈ 10^–6^ Ω, *R*_tr_ ≈ 350 Ω, *C*_MF_ ≈ 10^–7^ F, *C*_g_ ≈ 10^–7^ F, *R*_ion_ ≈ 600 Ω, and *C*_ion_ ≈ 6 × 10^–6^.

The normalized Bode and Nyquist plots (Figure 2b and c, respectively) reveal a progressive shift of resistive characteristics to lower frequencies as the mp-TiO_2_ thickness increases. Specifically, in the device with a 950 nm ETL thickness, the MF feature becomes more pronounced and completely decoupled from the first feature. This behavior is attributed to an increase in transport resistance due to reduced perovskite infiltration within the mesoporous structure. As the thickness reaches 1200 nm, this effect becomes the dominant phenomenon governing device behavior, ultimately masking the HF feature previously observed and leading the device to the PCE fall.

The appearance of the intermediate arch on the Nyquist plot (see Figure 2c) is related to a capacitive effect of the cell in the MF regime. The standard cell shows the typical capacitive response of a PSC, with two contributions, one in the HF regime and a second one in the LF regime; see the capacitance Bode plot in Figure 3a. This behavior has been previously modeled,^13^ atteibuting the capacitance at HF, independent of the applied voltage, to the geometrical capacitance (*C*_g_), which originates from the electric field formed between two opposing electrodes. Additionally, the capacitance at the LF is dominated by ionic behaviors and charge accumulation at interfaces within the device, strongly dependent on the applied voltage. This phenomenon arises from slow transient injected current, including slow movement of mobile ions, such as halide vacancies or organic cations, under an applied electric field.^20,21^ With an increment in mp-TiO_2_ up to 800 nm (see Figure 3b), a new feature at MF appears, which is not typically observed in PSCs. In this case, there is a low dependence on applied voltage. The MF feature becomes more pronounced in the sample with a thickness of 950 nm (see Figure 3c), with a clear dependence on applied voltage. The dependence of this feature on mp-TiO_2_ thickness and applied voltage indicates a chemical capacitance behavior^22,23^ produced by the progressive occupation of electronic states within the TiO_2_ mesoporous layer as the Fermi level of mp-TiO_2_ starts to approach the conduction band due to the electron injection from the perovskite, as in the case of a dye-sensitized solar cell^24,25^ (see Figure S2).

**Figure 3:**
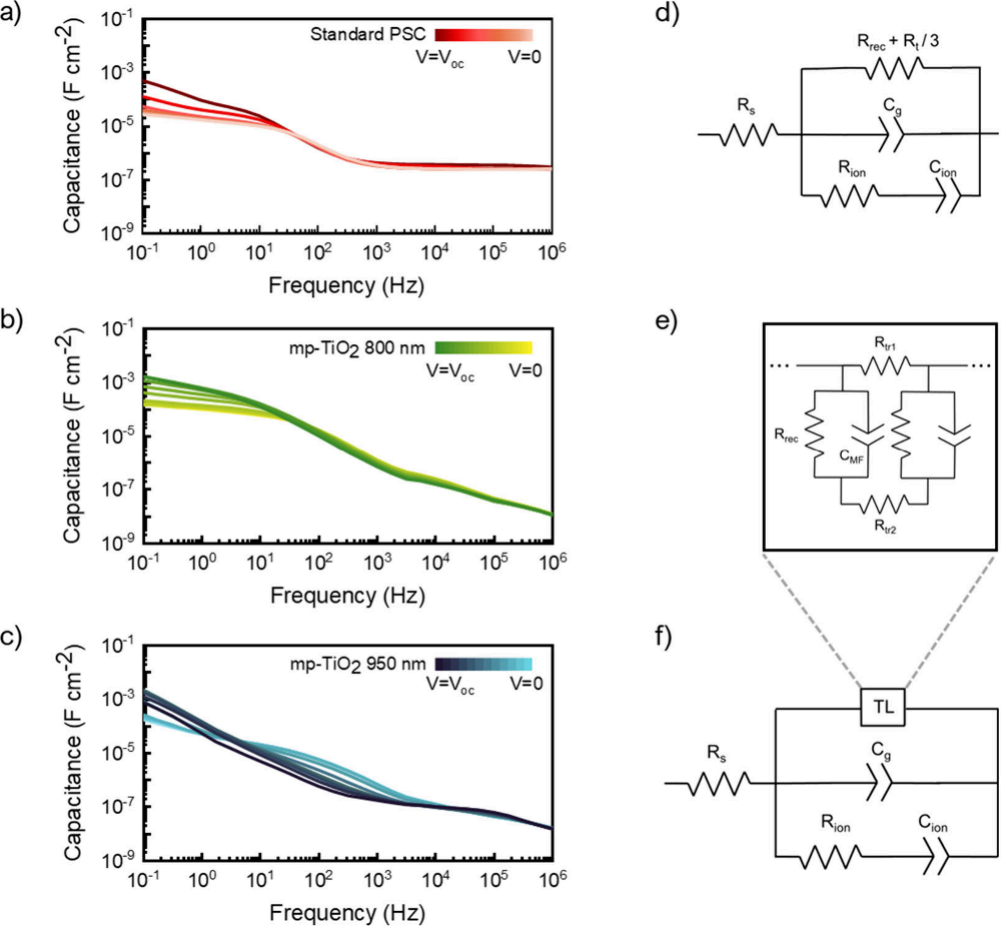
Bode plots of the (a) standard PSC and ITO-PSC with variating m-TiO_2_ thicknesses of (b) 800 and (c) 950 nm. The measurements were performed at different bias from *V*_app_ = *V*_oc_ to *V*_app_ = 0 V at 0.1 illumination conditions. Equivalent circuits used to fit the IS data for the (d) standard PSC and (e and f) ITO-PSC (see Figure 2 and Figure S3).

In a conventional thin-film PSC, the chemical capacitance of TiO_2_ is negligible because its value is significantly lower than *C*_g_ and *C*_ion_. However, a thicker mesoporous layer increases the level of electron injection into the TiO_2_ scaffold, leading to higher TiO_2_ chemical capacitance values. A similar phenomenon has been reported^13^ by varying perovskite absorber concentrations on the mesoporous structure, identifying a transition stage between the dye-sensitized impedance pattern (associated with a lower perovskite concentration) and a thin-film PSC pattern. It is important to emphasize that, in standard PSCs architectures, where thpical mp-TiO_2_ thicknesses and perovskite concentrations are employed standard PSC architectures, the mid-frequency capacitance is not observed, as charge transport is dominated by the perovskite material.

To accurately identify the relevant parameters, an equivalent circuit model is used to fit the IS data of three distinct samples: a standard PSC and two ITO-PSCs with mp-TiO_2_ thicknesses of 800 and 950 nm, respectively. Devices with thinner or thicker mp-TiO_2_ layers were excluded because they did not exhibit optimal photocurrent generation, resulting in unreliable spectra in a too short range of voltages. For the standard thin cell, the previously reported simplified model^13^ based on the parallel interaction of ionic and electronic processes (see Figure 3d) can be used. This model includes a series resistance, *R*_s_, linked to the electrodes and wiring, a geometrical capacitance, *C*_g_, and a resistor, *R*_rec+tr_ = *R*_rec_ + *R*_tr_/3, that combines both recombination, *R*_rec_, and all charge transport/injection processes, *R*_tr_. A parallel circuit branch comprising a resistance, *R*_ion_, and a capacitance, *C*_ion_, captures the ionic dynamics of the perovskite that dominates the LF region. This equivalent circuit has been successfully implemented in a wild range of PSC devices; however, it fails to accurately describe the IS spectra of systems exhibiting more than two distinct features, as is the case for the HTL-free samples analyzed. Fitting the ITO-PSC data requires an adaptation of such an equivalent circuit (see Figure 3d) that is used to describe the typical perovskite solar cells. The generalized equivalent circuit incorporates additional elements adapted from the transmission line (TL) model (see Figure 3e) used to analyze mp-based devices, such as DSSC.^26,27^ In particular, it considers the charge transport, recombination, and chemical capacitance of the mp-TiO_2_ layer independently (see Figure 3f). The modified equivalent circuit achieves remarkable agreement with the experimental IS data across the entire measured frequency range, as represented in Figure 2c and Figure S3. The fitted curves overlay the raw data points with minimal deviation, successfully reproducing both the three distinctive arches in the Nyquist plots and the MF capacitive effect observed in the ITO-PSC data displayed in Figure 3a–c.

The parameters extracted from the fitting to such an equivalent circuit are presented in Figure 4. *C*_g_, which dominates the high-frequency regime, is primarily determined by the composition and thickness of the absorbing material (Figure 4a). The behavior of this parameter is consistent with established perovskite solar cell characteristics, displaying the expected bias-independent response and an inverse relationship between *C*_g_ and the perovskite thickness. In the low-frequency domain, represented by the LF circuit branch, the increase in the mp-TiO_2_ thickness promotes the separation of perovskite domains within the mesoporous structure. This separation reduces ion mobility by obstructing the pathways for ion migration.^28,29^ This phenomenon is evidenced by an increase in both ion-associated *C*_ion_ and *R*_ion_ of the device^30^ (see Figure 4c and f).

**Figure 4:**
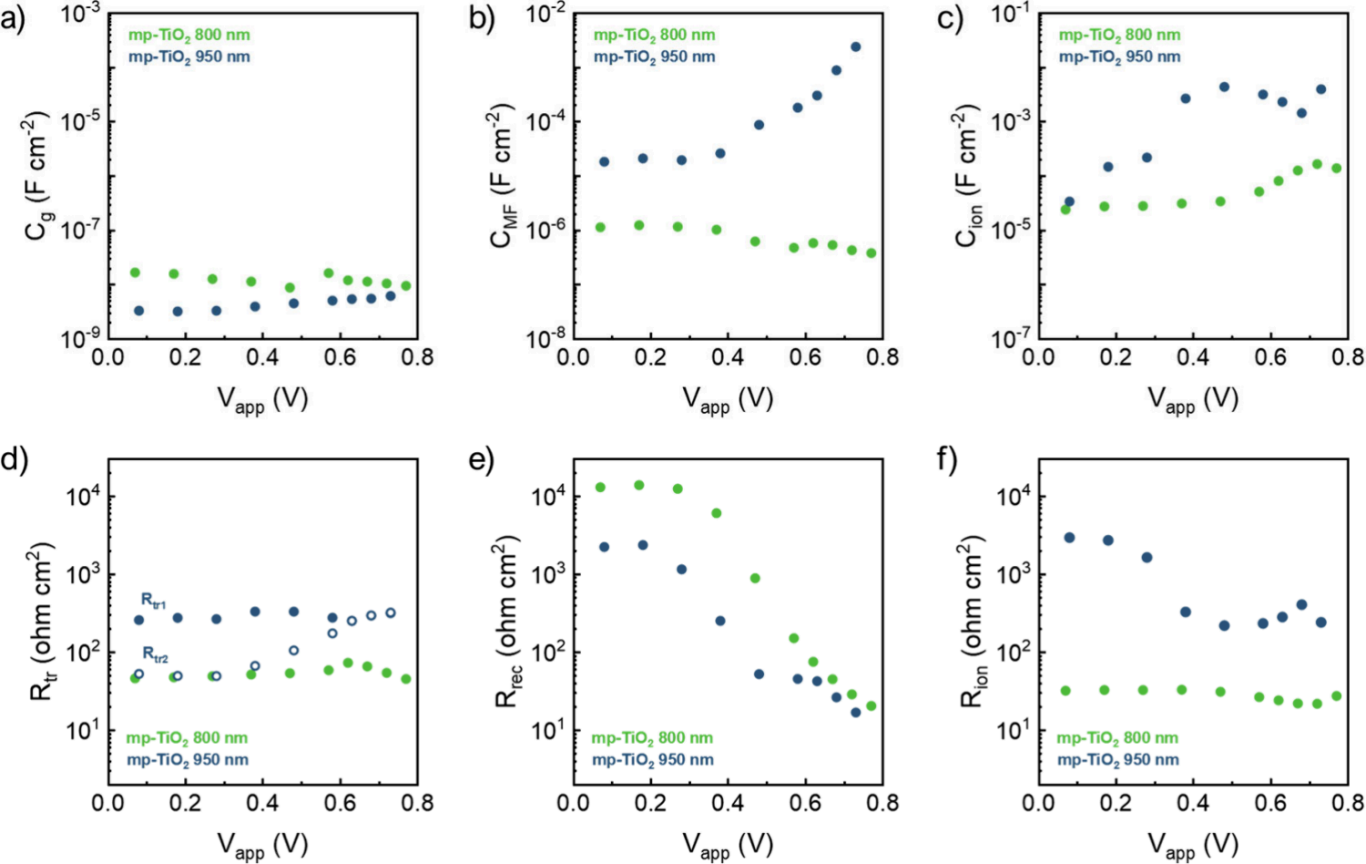
(a–c) Capacitive and (d–e) resistive elements as extracted from the fittings of the impedance spectra under 0.1 sun.

These modifications of the equivalent circuit allow identifying individually the chemical capacitance of the mp-TiO_2_ layer, charge transport, and recombination resistance. In particular, the incorporated capacitive element successfully captures the MF capacitive behavior observed in the Bode plot (Figure 3b and c) associated with the progressive occupation of electronic states within the TiO_2_ mesoporous layer. Notably, the exponential increase of this capacitance at higher voltages aligns well with a chemical capacitance behavior.^31^ It is particularly pronounced in devices with 950 nm ETL, as expected from its volumetric nature, and suggests non-negligible charge transport through the TiO_2_ mesoporous layer. Concurrently, the increment of the TiO_2_ thickness induces an increase of charge transport effects and significantly decouples electron and hole transport resistances. This effect originates from incomplete perovskite infiltration in thicker mesoporous structures, which limits vertical electron transport through the perovskite and consequently increases its transport resistance. As a result, the charge carrier injection from the perovskite to mesoporous TiO_2_ becomes a viable pathway for extraction to the FTO contact, similar to the case of dye-sensitized solar cells.^32^ This injection increases the TiO_2_ quasi-Fermi level, which is reflected in the exponential increase of chemical capacitance (see Figure 4b), characteristic of the exponential density of states in the band gap of mesoporous TiO_2_.^31^ The chemical capacitance follows an approximate exponential form at high potentials: *C*_μ_ = *C*_0_ exp­(α*qV*_F_/*k*_B_ *T*), where *k*_B_ *T* represents the thermal energy and *q* is the elementary charge. For a mp-TiO_2_ layer of 950 nm, we extract α = 0.34, in the range with reported values in the literature for mp-TiO_2_ in dye-sensitized solar cells.^33^ Overall, devices with thicker mesoporous TiO_2_ promote the charge transport through the ETL, which affects both resistive and capacitive parameters: it raises a chemical capacitance originated by the quasi-Fermi level of electrons in the mp-TiO_2_ layer; consequently, this results in higher and decoupled electron and hole transport resistances (see Figure 4d).

*R*_rec_ decreases with the thickness increment (see Figure 4e) and correlates directly with the *V*_oc_ losses in devices with the 950 nm mp-TiO_2_ layer.^34,35^ Note also that this device exhibits an anomalous behavior at higher voltages, characterized by two distinct *R*_rec_ slopes. This suggests the coexistence of multiple recombination mechanisms, highlighting the complexity of charge dynamics with thicker mesoporous structures. As a final validation of the equivalent circuit beyond fitting the measured data, it is key to ensure that the parameters used present unique physical meaning. Reconstructing the *j*–*V* curves from *R*_rec_ obtained through IS fitting^36^ can be a valuable approach to validating the recombinative origin of this parameter. This method utilizes the recombination extracted from the impedance and the electronic ideality factor (*m*) to recalculate the current value at any given voltage point. In devices with optimized charge extraction or in systems where charge recombination and transport can be decoupled into separate parameters (as in the case considered here), *j*–*V* should be reproducible using this approach, assuming no voltage dependence in the photogenerated current. Figure S4 depicts this curve reconstruction for devices with mp-TiO_2_ layers of 800 and 950 nm thickness. Experimental *j*_dc_ closely aligns with calculated points, confirming that the *R*_rec_ values extracted from the impedance fitting accurately represent the device’s actual recombination resistance. While the 950 nm mp-TiO_2_ device shows some localized discrepancies at higher voltages, where the *R*_rec_ vs *V*_app_ slope becomes less defined, the overall agreement remains strong. With the emergence of chemical capacitance in thicker devices and the implementation of the upgraded equivalent circuit, recombination and transport parameters that are usually coupled in planar PSCs can be identified independently. Consequently, the *j*–*V* reconstruction from IS data fitting becomes feasible even when charge transport effects are significant.

The IS response of the triple mesoporous ITO-based PSCs with ETL layer thickness varying from 500 to 1200 nm reveals an interplay between the mesoporous scaffold architecture and charge carrier dynamics, with a significant impact on resistive and capacitive features that cannot be captured by standard PSC IS models. Increasing the ETL thickness induces complex responses in the IS spectra, including the appearance of fingerprints typically observed in dye-sensitized solar cells. We propose an upgraded equivalent circuit model that enables the identification of these new features, incorporating parameters such as the chemical capacitance of the TiO_2_ layer that decouples the transport and recombination resistances. Our findings indicate that optimal thicknesses of mp-TiO_2_ can enhance device performance by improving electron injection and minimizing recombination losses. However, excessive thickness can hinder perovskite infiltration and degrade efficiency. In addition, the architecture and IS spectra response of ITO-based PSCs share a close resemblance with those presented by the perovskite carbon-based mesoporous devices, making our equivalent circuit model broadly applicable. Overall, these results underscore the importance of tailored mesoporous structures in optimizing scalable PSC architectures and provide a tool for their characterization.

## Experimental Methods

*Materials*. Hellmanex III detergent, Ti diisopropoxide bis­(acetylacetonate) (75 wt %, in isopropanol), polyvinylpyrrolidone (PVP, 55 000 and 10 000), PbI_2_ (99%), PbBr_2_ (99%), *N*,*N*-dimethylformamide (anhydrous 99.8%), acetic acid, and isopropyl alcohol (anhydrous 99.5%) were purchased from Sigma-Aldrich. TiO_2_ paste, MABr, and FAI were purchased from GreatCell Solar. Titanium­(IV) chloride (TiCl_4_) was purchased from Wako. Ethanol absolute (99.5%) and extra dry dimethyl sulfoxide (99.7%) were purchased from Acros. Indium tin oxide (nanopowder, <17–28 nm particle size) was purchased from Alfa Aesar. ZrO_2_ pastes were purchased from Solaronix. Chlorobenzene (99.8%, Sigma-Aldrich), tris­(2-(1*H*-pyrazol-1-yl)-4-tertbutylpyridine)­cobalt­(III) tri­[bis­(trifluoromethane)­sulfonimide] (FK209, Sigma-Aldrich), 30 NR-D transparent titania paste (GreatCell Solar Materials), bis­(trifluoromethane)­sulfonimide lithium salt (Li-TFSI, Sigma-Aldrich), ethanol (99.9%, Merck), spiro-OMeTAD (Lumtec), titanium diisopropoxide bis­(acetylacetonate) (TIAP, 75 wt % in isopropanol, Sigma-Aldrich), and 4-*tert*-butylpyridine (tBP 98%, Sigma-Aldrich) were used.

*Device Fabrication*. FTO-coated glasses were etched with laser and washed in an ultrasonic bath using soap, Hellmanex detergent (1% in water), and a mixture of ethanol and acetone. A compact TiO_2_ layer was deposited onto the substrates by spin coating with a 11.8% diisopropoxide bis­(acetylacetonate) solution in ethanol (5000 rpm, 30 s) after 20 min of oxygen plasma. The layer was annealed for 30 min in 450 °C. Next, a TiCl_4_ treatment was used by a bath (1.6 mL of TiCl_4_ in 150 mL of triple distilled water) for 30 min at 75 °C, followed by annealing on a hot plate for 30 min at 450 °C. Next, mp-TiO_2_ was fabricated using TiO_2_ paste (90 T). The thickness was modified by using different mesh sizes for the screen printing (130, 100, or 63). The 500 nm thick mp-TiO_2_ was fabricated by spin coating (5000 rpm, 30 s). mp-TiO_2_ was sintered at 500 °C for 30 min. A second TiCl_4_ treatment was carried out on mp-TiO_2_, followed by screen printing and annealing of the mp-ZrO_2_ layer (500 °C for 30 min). Finally, the ITO paste, prepared as reported in the past,^10^ was screen-printed with a 43 mesh and sintered in a furnace for 90 min at 590 °C.

On top of the complete mesoscopic stack, 1.6 μL of PbI_2_/PbBr_2_ solution (0.85:0.15, 1.4 M) was drop-casted and annealed in 70 °C for 30 min. Then, the substrate was immersed in a FAI/MABr solution (0.85:0.15, 0.06 M) for 30 min, then washed in isopropanol, and annealed for 2 h at 70 °C.

For the standard architecture, the TiO_2_ compact layer was deposited by spray pyrolysis with oxygen as the carrier gas. The TiO_2_ precursor solution was prepared by adding 1000 μL of titanium diisopropoxide into 9 mL of ethanol. The FTO substrates were heated to 450 °C before spraying and maintained at this temperature for 30 min after spraying. Afterward, a mesoporous TiO_2_ layer was deposited by spin coating (150 mg of 30-NRD titania paste dissolved in 1 mL of ethanol) at 4000 rpm for 20 s. Following deposition, the layer was dried at 100 °C and subsequently heated to 450 °C for 30 min. For the perovskite deposition, the substrates were transferred into a nitrogen-filled glovebox. The FAI/MABr solution was spin-coated at 2000 rpm for 10 s, followed by 5000 rpm for 30 s. At 25 s after the start, 400 μL of chlorobenzene was dripped onto the film as an antisolvent, and the films were annealed on a hot plate at 100 °C for 30 min. For the HTM, a 70 mM solution of spiro-OMeTAD with additives (tBP, 3.3 mol/mol spiro; LiTFSI, 0.5 mol/mol spiro; and FK209, 0.03 mol/mol spiro) was prepared in chlorobenzene. A total of 50 μL of spiro-OMeTAD solution was dynamically deposited by spin coating at 4000 rpm for 20 s with an acceleration of 800 rpm/s. Finally, 80 nm of gold was thermally evaporated under vacuum to serve as the top electrode.

*Device Characterization*. Current–voltage measurements were preformed using a solar simulator with a 450 W Xe lamp and output power of air mass coefficient of 1.5 global sunlight. The *j*–*V* curves were obtained by applying a varying external bias from 1.5 to −0.1 V on the cell and measuring the photocurrent with a Keithley model 2400 digital source meter.

Impedance spectroscopy was carried out using a Gamry 1010E potentiostat/galvanostat on complete solar cells under room conditions and illumination intensity of 10 mW cm^–2^. The measurements were performed at different offset voltages spaced 50 mV from open circuit to 0 V, with a 10 mV AC perturbation ranging between 1 MHz and 0.01 Hz. ZView software was employed to analyze the results and fit the data to the equivalent circuit. The solar cells were characterized in ambient conditions (*T* of ∼30 °C and RH of ≥60%) without any encapsulation, with an active area defined by a mask of 0.084 cm^2^ for standard PSC and 0.118 cm^2^ for HTL-free ITO-PSC devices.

Cross-sectional scanning electron microscopy measurements were performed with a S-4800 instrument from HITACHI (Tokyo, Japan), and a cross-sectional image of the ITO-PSCs was taken by a FEI Helios NanoLab 460F1 using a focused gallium ion beam to expose the layered structure.

## Supplementary Material



## Notes

Current–voltage characteristics of reference conventional cells, working mechanism schematics, extended impedance spectroscopy measurement data, and current–voltage characteristic reconstructions (PDF)
