# Supplementary material for: Working Mechanisms of Triple-Oxide Mesoporous Hole-Transport-Layer-Free Printable Perovskite Solar Cells via Impedance Spectroscopy
Source: J Phys Chem Lett. 2025 Aug 8;16(33):8410–7. doi: 10.1021/acs.jpclett.5c01405 (PMC12376095; doi:10.1021/acs.jpclett.5c01405)
Supplement: Supplementary file 1 [file jz5c01405_si_001.pdf]

Supplemental Information

Working Mechanisms of Triple-Oxide Mesoporous HTL-Free Printable Perovskite Solar Cells via Impedance Spectroscopy

Pablo F. Betancur[1], Maayan Sohmer[2], Iván Mora-Seró[3*], Lioz Etgar[2*], Pablo P. Boix[4*]

1. Instituto de Ciencia de los Materiales de la Universidad de Valencia (ICMUV), Paterna, València, Spain

2. Institute of Chemistry, Casali Center for Applied Chemistry, The Center for Nanoscience and Nanotechnology, The Hebrew University of Jerusalem, Jerusalem 91904, Israel.

3. Institute of Advanced Materials (INAM), Universitat Jaume I (UJI), Avenida de Vicent Sos Baynat, s/n, Castelló, 12071 Spain

4. Instituto de Tecnología Química, Universitat Politècnica València-Consejo Superior de Investigaciones Científicas, Av. dels Tarongers, València, Spain

* Corresponding Authors: sero@uji.es, lioz.etgar@mail.huji.ac.il, pablo.p.boix@itq.upv.es

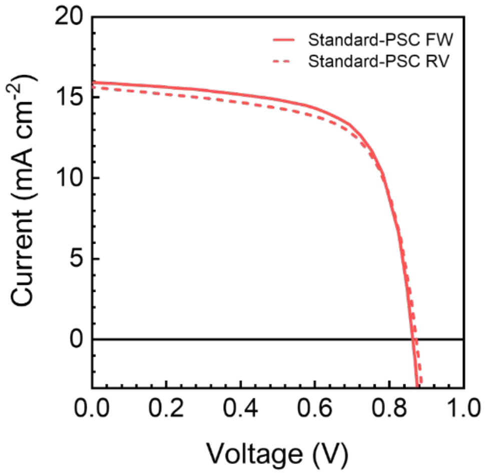

Figure S1. J-V curve of the analyzed standard PSC device with ITO/mp-TiO_2/perovskite/Spiro-MeOTAD/Au configuration at 1 sun illumination intensity.

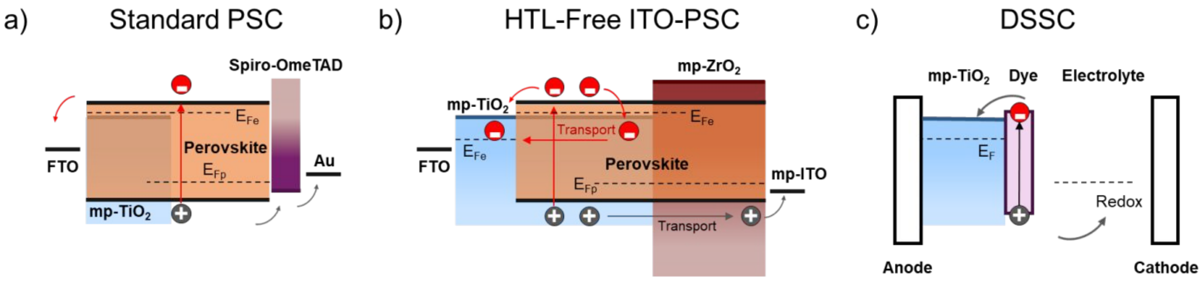

Figure S2. Basic energy band diagrams of (a) standard PSC, (b) ITO-PSC, and (c) dye-sensitized solar cell architectures under operating conditions. The diagrams show the photogeneration, transport, and extraction pathways for charge carriers. Energy levels of the constituent layers are depicted along with the quasi-Fermi levels for electrons and holes in the perovskite and mp-TiO_2 layers, where applicable to the charge transport processes.

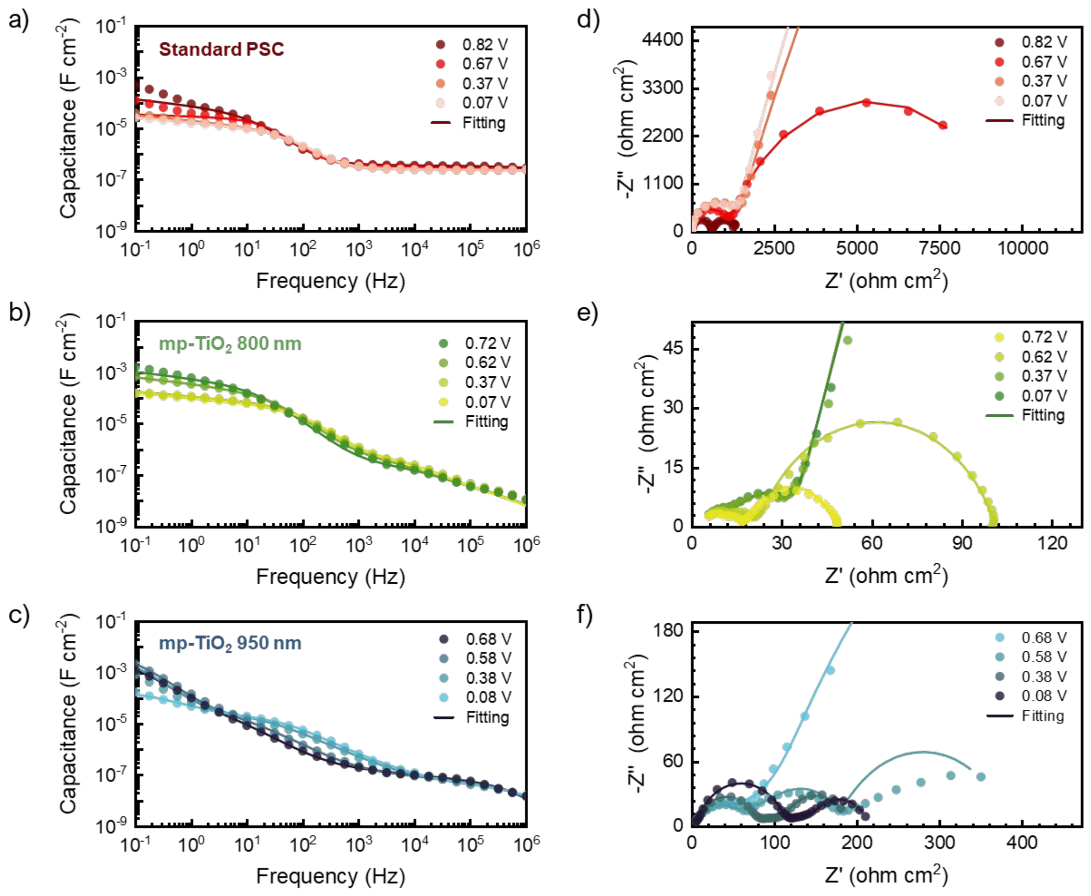

Figure S3. Comparison of experimental (dots) and fitted (lines) data using the equivalent circuits for (a-c) capacitance Bode plot and (d-f) Nyquist plot Bode of (a,d) the standard PSC and (b,e) the ITO-PSC with variating m-TiO_2 thickness, 800 nm and (c,f) 950 nm. Measured at various voltage biases at 0.1 sun illumination conditions.

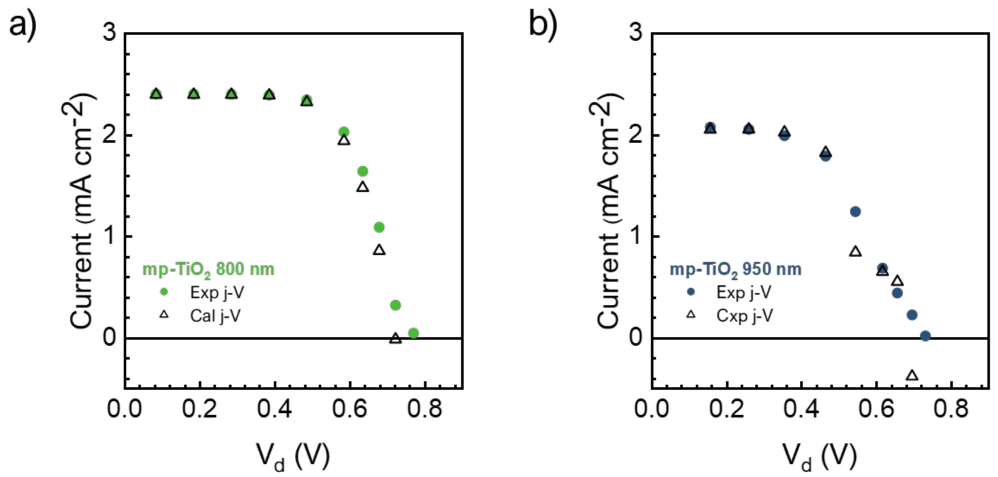

Figure S4. Experimental *j*−*V* curve (dots) and its reconstruction by using *m* value obtained from the slope of R_{rec} *vs* V_{app} (triangles). The voltage applied is optimized by the series resistance contribution as $V_d = V_{app} - (j_{R\text{-}s} + j_{R\text{-}tr})$.
